# Sentinel node mapping in thyroid cancer: an overview

**DOI:** 10.3389/fmed.2023.1163151

**Published:** 2023-06-20

**Authors:** Marco Puccini, Carlo Enrico Ambrosini, Leonardo Rossi, Luigi De Napoli, Gabriele Materazzi

**Affiliations:** ^1^Department of Surgical, Medical and Molecular Pathology and Critical Care Medicine, University of Pisa, Pisa, Italy; ^2^Endocrine Surgery Unit, Department of Surgery, University Hospital of Pisa, Pisa, Italy

**Keywords:** sentinel lymph node, thyroid cancer, medullary thyroid cancer, papillary thyroid cancer, radioguide, indocyanine green, lymph node dissection

## Abstract

In this paper we describe the current status of sentinel node mapping (SNM) in thyroid tumors and its potential perspectives. SNM in thyroid cancer has been tested since the end of the twentieth century, mainly in papillary thyroid cancer (PTC) and in medullary thyroid cancer (MTC). In PTC, it has been employed to find occult lymph node metastases in the central compartment of the neck as an alternative or indication for prophylactic dissection, by several methods. All of them have proven effective in spotting sentinel nodes, but the results have been somewhat diminished by uncertainty about the clinical significance of occult metastases in differentiated thyroid cancer. SNM in MTC has also been used to find occult lymph node metastases in the lateral compartments of the neck, also with excellent results hindered by a similar doubt about the real clinical significance of MTC micrometastases. Well designed, adequately sized randomized controlled trials are lacking, so SNM in thyroid tumors remains an interesting yet experimental methodology. New technology is emerging that could facilitate such studies, which could add solid information about the clinical significance of occult neck metastases in thyroid cancer.

## Introduction

Sentinel node mapping (SNM) is a widely used surgical technique to find subclinical lymph node metastases in apparently unaffected node basins in a variety of primary tumors. Here we report the current status of SNM in thyroid tumors and present our updated experience with it in medullary thyroid carcinoma.

## Sentinel node mapping in thyroid cancer

The sentinel node concept has been known since the second half of the twentieth century (1); the sentinel node is thought to represent the first lymphatic station draining from a primary tumor. This concept has been then applied to a variety of solid tumors, including thyroid tumors (2). The rationale for its use is to identify clinically occult metastases without resorting to a prophylactic lymph node dissection, which is associated with non-neglectable rates of complications and higher costs.

## The rationale for SNM in thyroid cancer

Preoperative evaluation of lymph nodal status in the neck relies on imaging, especially on preoperative high-frequency ultrasounds (US), which is, in experienced hands, effective in evaluating lateral compartments of the neck, but less so for the central compartment (3–5), which often is the first echelon of node metastasis from thyroid tumors. In his review, Xue et al. (3) reported that the accuracy of US is only 48.3%, while the sensitivity for predicting central lymph node metastasis is only 22.6–55%. The intraoperative exploration of neck compartments by the surgeon is also less than satisfying in detecting micrometastases (6, 7).

Since aim of thyroid cancer surgery is removal of the thyroid and affected lymph nodes, three alternative options exists for thyroid tumors with no clinical evidence of node metastases (cN0): (a) avoid dissection of nodes in favor of observation, with a certain risk of disease persistence/recurrence in case of occult metastases; (b) perform a prophylactic neck dissection, based on specific risk factors, with the risk of surgical overtreatment and related increase in morbidity; (c) SNM, with SN biopsy and therapeutic compartmental lymphadenectomy if the SN harbor a metastasis.

The concept of SNM has been excellently summarized by Dr. Expòsito Rodriguez: sentinel node mapping is the intraoperative complement of a negative US scan (8). In thyroid cancer, the SNM is a clue to accurate N staging while reducing surgical dissection, associated costs, and morbidity (9), while SNM has nearly null rates of related complications (10). A positive SNB could guide the extent of surgery, limiting lateral compartmental dissection only for therapeutic purposes (10–13) and personalize postoperative radiometabolic treatment (8, 14–17). Furthermore, SNM provides the ability to visualize altered lymphatic pathways and individual lymphatic patterns (18).

## The methods of SNM in thyroid cancer

The methods commonly used for harvesting the sentinel nodes in thyroid cancer can be schematized as follows:

(A) Visual tracers (e.g., blue dye, carbon nanoparticles and indocyanine green);
(B) Radioactive tracers (e.g., 99mTc saline solution, 99mTc-phytate and 99mTc-nanocolloid albumin);
(C) Hybrid tracers (e.g., nanocolloid albumin and methylene blue, indocyanine green and nanocolloid albumin); and
(D) Magnetic tracers, such superparamagnetic iron oxide (SPIO) nanoparticles (19, 20).

Besides, new tracers and techniques are currently under development and evaluation, such (68)Ga-tilmanocept PET/CT (21). These methods differ in the tracer employed, instruments necessary to identify the sentinel nodes, and logistics between the tracer injection and the surgical operation. Even studies using a similar method can differ in tracer amount and injection sites, timing from injection to optimal node spotting, and other variables. As a consequence, the comparison of the results between the various studies should be cautious, since small differences in detail could explain different results between the studies.

Nevertheless, some attempts have been made to compare the various techniques. Garau et al. (22) presented a meta-analysis, which included 45 studies, comparing 4 kind of procedure: vital-dye (VD) alone, Tc-nanocolloid planar lymphoscintigraphy with the use of intraoperative hand-held gamma probes (LS), both Tc-nanocolloid planar lymphoscintigraphy with intraoperative gamma probe and VD (LS + VD), Tc-nanocolloid planar lymphoscintigraphy with preoperative SPECT/CT, and intraoperative gamma probe (LS-SPECT/CT). Tc nanocolloid was superior to methylene blue, and the addition of SPECT/CT improved identification of metastatic SLNs outside the central neck (22). Gelmini et al. (23) conducted a prospective, non-randomized study comparing blue dye SNM (40 patients), lymphoscintigraphy (5 patients) and the combined technique (40 patients) with apparently better results with the single tracer techniques. In the recent years some surgeons have abandoned patent blue dye in favor of nano colloidal albumin: blue dye is economically advantageous and does not need a Nuclear Medicine facility, but is more difficult to manipulate intraoperatively since it can easily stain the operative field, gloves, swabs, parathyroid glands, and finally hide from sight the recurrent laryngeal nerve. Radioisotope SNM, on the other hand, localizes better SN in the lateral compartments, does not fix in parathyroid glands and does not blur the vision of operative field, so it is preferred, when available.

A new technical adjunct to limit the false negative rate of SNM on frozen sections is one-step nucleic acid amplification (OSNA), which allows real-time (intraoperative) detection of mRNA encoding for cytokeratin 19; OSNA has been tested for papillary thyroid carcinoma (24, 25), with satisfying results.

### The results of SNM in differentiated thyroid cancer

Overall, although large prospective RCT are lacking, data reported in the literature attest that SNM is an effective method for intercepting most clinically occult metastases in approximately 30–40% of cases (8, 10–13, 15–17, 22, 23), both in the central and lateral neck, so that SNM could serve as an objective indicator for RAI or to modulate radioactivity doses, or for therapeutic compartmental dissection. The controversy arises when we look at the clinical usefulness of SNM, since no differences appear in observed relapse rates and disease specific survival when applying SNM, prophylactic dissection, or simple observation (26). We have tested SNM in a more aggressive subset of differentiated thyroid carcinomas, those harboring a V600E BRAF mutation (27). Even in this group with a potentially higher loco-regional relapse rate, we observed no structural or biological relapse after a long follow-up (the current mean follow-up is 7 years).

Opponents of SNM in thyroid cancer report a high false negative rate compared with prophylactic dissection (5); another estimation study argued that the higher costs for implementing SNM outweighed potential savings (28).

### SNM in medullary thyroid cancer

We found it particularly interesting to apply SNM in medullary thyroid cancer (MTC) for several reasons: MTC is a lymphophilic tumor with early nodal spread; the most effective therapy is initial surgery, while RAI has no effect on this C-cell derived tumor; and persistence of disease can be detected by a specific circulating marker, calcitonin (CT), which can also reveal minimal residual disease after calcium infusion stimulation. Since central compartment dissection is considered an integral part of initial surgery even for cN0 cases (29, 30), SNM in MTC applies to lateral compartments of the neck, for which a prophylactic dissection remains controversial (30). Some surgeons perform lateral dissection according to one or more risk factors for the presence of lateral neck metastases form MTC, such preoperative CT levels, tumor diameter, presence and number of central compartment nodal metastases, postoperative basal and stimulated CT levels, and histologic features of the primary tumor including desmoplastic reaction; however most surgeons consider lateral dissection only in the presence of clinically evident metastatic nodes, since prophylactic lateral dissection carries a potential risk of surgical complications and related morbidity from unnecessary LND. A recent study comparing prophylactic lateral neck dissection vs. no lateral dissection in cN0 MTC patients with CT levels > 200 pg/ml found that prophylactic dissection is not associated with improved survival (31).

### The results of SNM in medullary thyroid cancer

We found only two studies on SNM for cN0 MTC, besides ours (9, 32, 33). In this paper we report an update of our cases. All three studies are limited in number of patients, and used different methods for SNM: two centers used a radiotracer (Pisa: 99mTc-nanocolloid-albumin; Seoul: 99mTc-Phytate), while Belgrade used blue dye. Tumor characteristics were relatively similar. The sentinel node identification rate was excellent, but the rate of metastatic SN was higher in Pisa. The biopsy was analyzed by definitive histology in Pisa, and by frozen section in the other two centers. After the biopsy, the surrounding tissue (the level in which the sentinel node was detected) was also removed in Pisa and Belgrade (in case of negative SN on frozen section), the whole compartment in case of metastatic sentinel at frozen section in Belgrade, while in Seoul the biopsy was always followed by a lateral compartment dissection, regardless of frozen section results. The studies differ also in terms of non-sentinel metastatic nodes and in central compartment metastases (Table 1). Of note, in all cases, when the sentinel node was non-metastatic, no other metastases were found in the lateral neck (as in the two Korean cases where a sentinel node was not detected).

**TABLE 1:** Studies evaluating sentinel node mapping (SNM) of lateral compartment in medullary thyroid cancer – methods.

	Tumor characteristics, basal CT, tumor diameter	Tracer	Injection	Timing of surgery	Pts (% SDR)	SNB assessment	Surgery after SN biopsy
Santrac (33) Belgrade	microMTC cN0M0 CT 234 (7, 2–637) Ø 8 mm (3–10)	Methylene blue	Peritumoral	Few minutes	20 (100%)	FS + histology	If SN negative: enlargement of dissection (in the area of removed SN) If SN positive: therapeutic lateral dissection
Kim (9) Seoul	MTC cN0M0 CT 248 (49–1049) Ø 15 mm (IQR 6–20)	Tc^99m^-Phytate	Intratumoral echo-guided	5 h later	16 (87.5%)	FS + histology	Prophylactic lateral dissection
Puccini (32) Pisa	MTC cT1N0M0 CT 103 (40–142) Ø 9.8 mm (6–14)	Tc^99m^-nanocolloid	Intratumoral echo-guided	2–6 h later	7 (100%)	Histology	Enlargement of dissection (in the area of removed SN)

CT, plasma calcitonin; Pts (SDR%), number of patients (sentinel node detection rate); SNB, sentinel node biopsy; MTC, medullary thyroid cancer; FS, frozen section; Ø, diameter.

The effects of these studies are shown in Table 2: there is no evidence of relapse even at the longer follow-up in our series (mean, 110 months). CT and CEA levels are undetectable both in Pisa and in Belgrade, while Kim et al. (9) report low but detectable (probably non-stimulated) CT levels for 9 patients with a mean follow-up of 3 years. This should be evaluated with caution, but it is not clearly representative of a residual disease. In our series, the original plan was to practice a therapeutic lateral dissection in case of metastatic sentinel node, but until now no patient has undergone salvage surgery since biological evidence of relapse is lacking (according to basal and stimulated CT levels). We registered one death due to the rupture of a cerebral aneurism. In these limited experiences, no occult metastases were detected when the sentinel node was tumor-free.

**TABLE 2:** Studies evaluating SNM of lateral compartment in medullary thyroid cancer – results.

	Central compartment metastases at histology Nr patient (%)	Lateral SN metastases at histology Nr patient (%)	Lateral compartment status in SN positive pts	Lateral compartment status in SN negative pts	Mean follow-up (mean in months)	Biochemical disease relapse (CT, CEA)	Structural relapse (US)
Santrac (33) Belgrade	2 (10%)	2 (10%)	Further metastases found in both cases	No metastases	80 mo	No	No
Kim (9) Seoul	4 (25%)	3 (21%)^(1)^	No further metastases	No metastases^(2)^	34 mo (2 lost at f-up)	No^(3)^	No
Puccini (32) Pisa	1 (14%)	3 (43%)	No further metastases	No metastases	110 mo (1 death for unrelated causes)	No	No

SN, sentinel node; CT, calcitonin; CEA, carcinoembriogenic antigen; US, neck ultrasounds; mo, months.

^(1)^At frozen section, one of them was labeled as negative.

^(2)^Including two patients with negative SNM.

^(3)^In nine patients with at least 3 years follow-up mean CT level was 7.7 pg/ml.

The considerations regarding SNM in medullary thyroid cancer are surprisingly similar to those regarding differentiated cancer: SNM is very efficient in identifying occult metastases in the lateral compartment, and apparently if the sentinel nodes are unaffected, the risk of occult metastases in other nodes seems extremely low. The data published suggest that SNM of the lateral compartments with therapeutic dissection only in case of positive sentinel node could be equivalent to prophylactic dissection, but the limited volume of patients tested and the structure of the studies represent strong bias and prevent any conclusions, and impose the need for prospective, randomized multicenter studies with a standardized technique and a sufficient statistical power to eventually include SNM in the guidelines for surgical management of MTC.

In conclusion, SNM for thyroid cancer remains an interesting yet experimental methodology, which has been proven to be safe and effective in identifying occult metastases, and has potential applications whose clinical impact has still to be demonstrated.

The ideal method should be economically and logistically affordable, relatively easy to practice, with a quick learning curve, the identification of sentinel nodes should have minimal interobserver variation, and should be safe for patients. It should be non-inferior to prophylactic resections, and superior to simple observation, especially in terms of long-term oncologic outcomes.

The continuing advances in technology are offering new tracers and methods for performing practical and cost-effective SNM, but the main challenge remains to demonstrate a consistent impact on the clinical management of thyroid cancer patients.

## Data availability statement

The original contributions presented in this study are included in the article/supplementary material, further inquiries can be directed to the corresponding author.

## Author contributions

MP: concept and writing of the manuscript. CA and LD: corrections and data extraction. LR: literature search. GM: supervision and critical revision. All authors contributed to the article and approved the submitted version.
